# Dengue Virus Serotype 2 Intrahost Diversity in Patients with Different Clinical Outcomes

**DOI:** 10.3390/v13020349

**Published:** 2021-02-23

**Authors:** Maria Celeste Torres, Marcos Cesar Lima de Mendonça, Cintia Damasceno dos Santos Rodrigues, Vagner Fonseca, Mario Sergio Ribeiro, Ana Paula Brandão, Rivaldo Venâncio da Cunha, Ana Isabel Dias, Lucy Santos Vilas Boas, Alvina Clara Felix, Maira Alves Pereira, Luzia Maria de Oliveira Pinto, Anavaj Sakuntabhai, Ana Maria Bispo de Filippis, on behalf of ZikAction Consortium

**Affiliations:** 1 Laboratório de Flavivírus, Instituto Oswaldo Cruz, Fiocruz, 21040-360 Rio de Janeiro, Brazil; marcosclm@ioc.fiocruz.br (M.C.L.d.M.); cintia_damasceno7@yahoo.com.br (C.D.d.S.R.); ana.bispo@ioc.fiocruz.br (A.M.B.d.F.); 2 KwaZulu-Natal Research Innovation and Sequencing Platform (KRISP), School of Laboratory Medicine and Medical Sciences, Nelson R Mandela School of Medicine, College of Health Sciences, University of KwaZulu-Natal, 4041 Durban, South Africa; vagner.fonseca@gmail.com; 3 Laboratório de Genética Celular e Molecular, Instituto de Ciências Biológicas, Universidade Federal de Minas Gerais, 31270-901 Belo Horizonte, Brazil; 4 Coordenação Geral dos Laboratórios de Saúde Pública/Secretaria de Vigilância em Saúde, Ministério da Saúde, (CGLAB/SVS-MS) Brasília, 70719-040 Distrito Federal, Brazil; 5 Superintendência Secretaria de Vigilância em Saúde do Estado do Rio de Janeiro, 20031-142 Rio de Janeiro, Brazil; mario.ribeiro@saude.rj.gov.br; 6 Laboratório Central Noel Nutels/LACEN, 20231-092 Rio de Janeiro, Brazil; ana.brandao@saude.rj.gov.br; 7 Coordenação de Vigilância em Saúde e Laboratórios de Referência da Fundação Oswaldo Cruz, FIOCRUZ, 21040-360 Rio de Janeiro, Brazil; rivaldo.cunha@fiocruz.br; 8 Instituto de Medicina Tropical, Faculdade de Medicina, Universidade de São Paulo, 05403-000 São Paulo, Brazil; abela.ana@gmail.com (A.I.D.); lucyvilas@yahoo.com.br (L.S.V.B.); clarafelixx@gmail.com (A.C.F.); 9 Fundação Ezequiel Dias/LACEN, 31630-903 Belo Horizonte, Brazil; maira.pereira@funed.mg.gov.br; 10 Laboratório de Imunologia Viral, Instituto Oswaldo Cruz, Fiocruz, 21040-360 Rio de Janeiro, Brazil; lmopnogueira@gmail.com; 11 Functional Genetics of Infectious Diseases, Department of Global Health, Institut Pasteur, 75015 Paris, France; anavaj.sakuntabhai@pasteur.fr

**Keywords:** dengue virus, serotype 2, intrahost diversity, severe disease

## Abstract

Intrahost genetic diversity is thought to facilitate arbovirus adaptation to changing environments and hosts, and it might also be linked to viral pathogenesis. Dengue virus serotype 2 (DENV-2) has circulated in Brazil since 1990 and is associated with severe disease and explosive outbreaks. Intending to shed light on the viral determinants for severe dengue pathogenesis, we sought to analyze the DENV-2 intrahost genetic diversity in 68 patient cases clinically classified as dengue fever (*n* = 31), dengue with warning signs (*n* = 19), and severe dengue (*n* = 18). Unlike previous DENV intrahost diversity studies whose approaches employed PCR, here we performed viral whole-genome deep sequencing from clinical samples with an amplicon-free approach, representing the real intrahost diversity scenario. Striking differences were detected in the viral population structure between the three clinical categories, which appear to be driven mainly by different infection times and selection pressures, rather than being linked with the clinical outcome itself. Diversity in the NS2B gene, however, showed to be constrained, irrespective of clinical outcome and infection time. Finally, 385 non-synonymous intrahost single-nucleotide variants located along the viral polyprotein, plus variants located in the untranslated regions, were consistently identified among the samples. Of them, 124 were exclusively or highly detected among cases with warning signs and among severe cases. However, there was no variant that by itself appeared to characterize the cases of greater severity, either due to its low intrahost frequency or the conservative effect on amino acid substitution. Although further studies are necessary to determine their real effect on viral proteins, this heightens the possibility of epistatic interactions. The present analysis represents an initial effort to correlate DENV-2 genetic diversity to its pathogenic potential and thus contribute to understanding the virus’s dynamics within its human host.

## 1. Introduction 

Dengue fever (DF) is the arboviral disease with the strongest impact in terms of morbidity and mortality worldwide. Latest estimations reported that annually 390 million people around the world get infected by dengue virus (DENV), of which 96 million ultimately manifest the disease [1]. DENV infection can range from asymptomatic infection to a debilitating and potentially life-threatening acute disease in human hosts [2]. Antibody-dependent enhancement (ADE) phenomenon explains why certain cases progress to severity [3]. However, as many other hemorrhagic dengue cases occur during primary DENV infections, ADE might not be a necessary condition for the development of disease severity, which ultimately raises the question about the role of viral factors in the infection process [4]. DENVs are positive-sense, single-stranded RNA viruses that belong to the *Flaviviridae* family, genus *Flavivirus*. The DENV genome size is approximately 10.7 kb and contains a region coding for a single polyprotein flanked by a short 5′ untranslated region (UTR) and a longer 3′ UTR, both highly structured and carrying elements essential to the virus replication. The polyprotein is post-translationally cleaved into three structural proteins (capsid (C), pre-membrane/membrane (preM/M), and envelope (E)) and seven non-structural proteins (NS1, NS2A, NS2B, NS3, NS4A, NS4B, and NS5) [5]. NS5 encodes for the replicative RNA-dependent RNA polymerase, which is a low-fidelity enzyme and thus prone to introduce genetic variability into the viral population during each cycle of RNA replication. Consequently, new viral variants get continuously generated within a single host, shaping what is defined as ‘intrahost diversity’ [6]. Considering that it was demonstrated for RNA viruses that just one or a few amino acid replacements within a single protein are enough to modify a particular biological feature of the virus [7,8], the intrahost diversity takes a place of high relevance on the study of the evolution of DENV populations during the course of human infection, and its relation to disease severity. Intrahost genetic diversity is thought to be advantageous for RNA viruses by facilitating their adaptation to changing environments and hosts, and as was demonstrated for many other viruses [9,10,11,12], might also influence their pathogenicity.

DENV-2 genotype III (previously named as the Asian/American genotype [13]) is circulating in Brazil since 1990 [14]. In 2008, a DENV-2 outbreak was associated with increased disease severity and a high mortality rate [15,16]. In this context, and to better understand the association of viral features with severe dengue pathogenesis, we explored DENV-2 intrahost genetic diversity in Brazilian patients with different clinical outcomes during the calendar period 2008–2019. 

Several investigations attempted to determine the correlation between DENV intrahost diversity and disease severity, employing different experimental designs—from PCR+cloning+Sanger sequencing of the E gene [17,18] to PCR+Next-generation sequencing of the complete genome [19,20]. However, all of these studies employed PCR as a molecular tool, potentially generating mutational bias through amplification and primer mismatching. Our study’s experimental approach consisted in an amplicon-free deep-ranging coverage of the viral genome, aiming to most reliably reflect the viral genetic variability at each stage of patient infection, to finally assess a correlation with disease severity.

## 2. Methodology 

### 2.1. Ethical Statement

This study was approved by the Oswaldo Cruz Institute Ethical Committee in Research (CAAE 90249219.6.1001.5248 number 2.998.362). All methods were performed in accordance with the World Medical Association Declaration of Helsinki.

### 2.2. Study Samples

Sixty-eight serum samples of DENV-2 confirmed cases from the Brazilian states of Rio de Janeiro, São Paulo, and Minas Gerais, collected between 2007 and 2019 were included in our study. Samples were sent to our laboratory by spontaneous demand for diagnostic purposes, accompanied by their respective epidemiological sheets but with patients’ identification already encoded. The cases analyzed here were clinically classified according to the 2009 World Health Organization guidelines [2]. 

### 2.3. Immune Response Classification

An in-house ELISA assay was employed to titer anti-DENV-2 IgGs. IgG titer was correlated with patients’ days of symptoms to determine the primary or secondary character of the immune response against DENV (See Table S7), as described by Miagostovich et al. in 1999 [21].

### 2.4. Viral RNA Isolation and Quantification

Total RNA was extracted from 140 µL human sera samples employing the QIAmp Viral RNA Mini kit (Qiagen, Hilden, Germany), under the manufacturer’s instructions. Extracted RNA (5 µL) was quantified by real-time RT-PCR on an AriaMx Thermal cycler (Agilent Technologies, Santa Clara, CA, USA), following protocols described by Santiago et al. in 2013 [22]. For the RNA quantification, a standard calibration curve was constructed using serial dilutions of RNA extracted from the DENV-2 strain 40247 [23], with an initial concentration of 5.01 × 10^5^ pfu (plaque-forming unit)/mL.

### 2.5. Virus Genome Deep Sequencing

An initial clarification step was performed for samples 160–209 by centrifuging sera at 3000× *g* over 30 min at 5 °C. Subsequently, the supernatants were passed through 0.22 µm filters to remove bacterial cell-sized particles and other particulate debris. An aliquot of 140 µL of these filtered sera, plus remaining serum samples (137–159) was supplemented next with 0.1 M β-mercaptoethanol, and total RNA was extracted as described in the previous section, with the exception that the final elution was carried out with 20 μg/mL Linear Acrylamide in RNase-free water (H_2_O-LA). Extracted nucleic acids (60 µL) were immediately treated with four units of Turbo DNase (Life Technologies, Carlsbad, CA, USA) to digest DNA contaminants, and were purified with Agencourt RNA XP beads (Beckman Coulter Genomics, Chaska, MI, USA), with a final elution in 30 µL of H2O-LA. 

Selective depletion of human ribosomal (rRNA) and carrier RNA from viral RNA samples, randomly-primed cDNA synthesis, and library construction were performed, as described previously [24], with slight differences for samples 160–209: (i) the dnased-RNA samples were treated with a non-thermostable RNase H (New England Biolabs, Ipswich, MA, USA) for selective contaminant RNA depletion, employing the commercial enzyme buffer and performing the reaction at 37 °C instead of 45 °C; (ii) cDNA synthesis was performed using the Superscript-IV reverse transcriptase system (Invitrogen), reducing the first-strand synthesis reaction time from 50 to 10 min; (iii) libraries were constructed by employing the Illumina DNA Prep kit (formerly named Nextera DNA Flex) with reaction volumes reduced to half the recommended, to reduce tagmentation and number of integration sites. Eighteen cycles of libraries PCR amplification were performed, following the manufacturer’s cycling conditions. Finally, the multiplexed libraries’ yield was determined with an ‘Agilent High Sensitivity DNA kit’ in a Bioanalyzer 2100 (Agilent Technologies, Santa Clara, CA, USA) and quantified with a Qubit fluorometer (Life Technologies, Carlsbad, CA, USA). Libraries were pooled in equimolar concentrations and paired-end sequenced in one of the Illumina’s system: Nextseq500 (75 cycles; samples 137–159) at the Institut Pasteur, France, or HiSeq4000 (300 cycles; samples 160–209) at the Novogene company, EUA. 1–5% PhiX library was employed in both cases as a control for Illumina sequencing runs.

Two DENV-2 negative human sera were processed and sequenced together with the samples to discard potential cross-contamination among samples. Additionally, two libraries were constructed from synthetic commercial plasmids (pGEM-3Zf, Promega, and pUCIDT-Kan, Integrated DNA Technologies) to assess the potential artefactual errors introduced by libraries PCR amplification and sequencing.

### 2.6. Bioinformatics Data Processing 

Sequencing reads were demultiplexed using bcl2fastq v2.15.0 (Illumina). Paired-end reads from each sample were first trimmed by removing Illumina adapter sequences and bases of low quality using Trimmomatic v0.36, with the following settings: ILLUMINACLIP:NexteraPE-PE.fa:2:30:10:5:true LEADING:3 TRAILING:3 SLIDINGWINDOW:4:15 MINLEN:20 [25]. Next, depletion from human contaminants was carried on with BMTagger (ftp://ftp.ncbi.nlm.nih.gov/pub/agarwala/bmtagger/) and BLASTN [26], and the reads were then filtered to DENV-2 serotype using LASTAL [27] and JX669477/JX073928 (GenBank accession numbers) as the reference sequences. The resulting reads were de novo assembled by employing Trinity [28], and the contigs were scaffolded and refined with MUMMER [29] and MUSCLE [30]. The de novo assembled consensus sequence was then employed as a reference where trimmed + filtered reads were mapped on to, using Novoalign (http://www.novocraft.com/products/novoalign/). The above software (except Trimmomatic) was implemented in a publicly available pipeline described by Park et al., 2015 [31] and run under the default settings. To limit the influence of PCR artefacts, duplicated reads were thereafter removed with Picard’s MarkDuplicatesWithMateCigar tool (http://broadinstitute.github.io/picard/) (with settings: --REMOVE_DUPLICATES = true --AS = true --SKIP_PAIRS_WITH_NO_MATE_CIGAR = true), and the Genome Analysis Toolkit (GATK, https://software.broadinstitute.org/gatk/) was employed to identify variant positions and realign reads around insertion/deletion (indels) positions (IndelRealigner option). Finally, quality scores were added to indels positions with Lofreq v.2.1.3.1 (option indelqual), and intrahost variants [iSNVs and iLVs (including deletions and insertions)] and their proportion among all DENV-2 sequencing reads were called with the same software, by only considering alternate bases with Phred quality equal to or higher than 30 (-Q 30) [32]. Variant calls with a significant strand bias (*p* < 0.05) and a frequency lower than 0.5% were removed from the dataset obtained for each sample (cut-off value inherently obtained from the variant-calling analysis of both the commercial plasmids and the PhiX sequencing control). Moreover, for samples with a low coverage depth (i.e., <100X), variants passing the above filters but present in only one forward and reverse reads (2 reads total) were also discarded to diminish the errors inherently associated with samples’ processing and NGS. A final manual edition on the consensus sequences was carried out by considering the variants passing all filters but presenting an intrahost frequency higher than 50%. Samtools v1.9 [33] was employed as additional software to convert, sort, and index files. 

After human sequence depletion and filtering to only keep the DENV-2 sequences, fastq files for all sequences generated in this study were deposited in the NCBI-Sequence Read Archive under the BioProject ID PRJNA541495. 

### 2.7. Phylogenetic Analysis

Whole-genome consensus sequences obtained by deep-sequencing were aligned using Mega v7.0 [34] to 321 DENV-2 sequences available in GenBank (https://www.ncbi.nlm.nih.gov; as retrieved on 10 September 2020, keeping only the full genome sequences (GenBank Accession numbers can be found in Table S8). Sequence alignment was next manually curated to remove the artefacts. Evidence for potential recombination events in the alignment consensus sequence was discarded (Phi-test; *p* = 0.3427) with the Recombination Detection Program v.4.101 (http://web.cbio.uct.ac.za/~darren/rdp.html). Maximum likelihood phylogenetic trees were constructed using the RaxML v8.2.8 software [35] with 1000 bootstrap replicates, under the GTR + I+G substitution model obtained by Akaike’s information criterion and likelihood value in Jmodeltest v2.1.6 [36]. The consensus tree was visualized in FigTree v1.4.4 (http://tree.bio.ed.ac.uk/software/figtree/).

### 2.8. Intrahost Viral Genetic Diversity Assessment

To analyze the existence of patterns of DENV-2 intrahost diversity in samples, according to patient’s clinical classification, the iSNV/LVs profile obtained after variant calling was first assessed for each sample. Since the variant-calling procedure returned each minor variant’s position in the complete viral genome (nucleotides 1 to 10723), their specific position on each particular viral gene was computed by employing an in-house script developed in R v3.5.3 (https://www.r-project.org/). Next, the impact of iSNV/LVs on each viral gene and amino acid encoding protein was obtained with the SnpEff software v4.3 [37]. iSNV/LVs identity, frequency, position, and impact over viral protein were then assessed for each clinical classification category, normalizing by group size to avoid biasing. The same procedure was carried on for the cases clustered by the immune response.

### 2.9. Natural Selection Assessment

The magnitude and direction of intrahost natural selection were estimated with the ratio of non-synonymous (dN) to synonymous (dS) substitutions per non-synonymous and synonymous coding sequence sites, using the Jukes-Cantor formula, as described elsewhere [38]. The dN/dS ratio represents a measure of selective pressure; thus, a ratio dN/dS > 1 results when changes in the protein sequence are favored by natural selection (evidence of positive selection), while a ratio < 1 is expected if natural selection suppresses protein changes (evidence of negative selection). A dN/dS ratio equal to 1 represents a situation of neutral evolution [38]. The DnaSP software v6.12 [39] was employed to obtain the number of synonymous and non-synonymous sites for each sample consensus sequence. 

### 2.10 Statistical Analysis

Statistical comparisons were performed using the GraphPad Prism v7.0 (https://www.graphpad.com/scientific-software/prism/).

## 3. Results 

### 3.1. Samples Characteristics 

Sixty-eight DENV-2 cases were clinically classified according to the WHO Guidelines [2] as follows—(a) dengue fever (DF) (45.6% of total cases, *n* = 31), (b) dengue with warning signs (WS) (27.9% of total cases, *n* = 19), and (c) severe dengue (SD) (26.5% of total cases, *n* = 18). Two and twelve WS and SD cases, respectively, were identified as fatal cases. Cases presenting a primary infection were 57.4% (*n* = 39), while the remaining 42.6% (*n* = 29) presented a secondary infection. When comparing the patient’s viral load at the time the samples were collected, the DF cases presented a higher median concentration (Table 1; Kruskal-Wallis test, *p* < 0.0001). When cases were grouped by the days since the onset of symptoms, viral load median also resulted higher for samples taken up to two days since the onset of symptoms, as expected, compared to those with three days or more (Kruskal-Wallis test, *p* = 0.0002). Indeed, in this cohort, clinical classification correlated strongly with patients’ days of symptoms (Pearson r correlation, *p* < 0.0001; Table S1), consistently with previous findings [40]. On the other hand, however, no statistic-supported association was detected between clinical classification and patients’ immune response (Table S1). As well, viral load medians did not differ statistically among cases with a primary or secondary immune response (median [interquartile range (IQR)]: 2.61 × 10^4^ [1.06 × 10^2^–5.17 × 10^4^] and 7 × 10^3^ [2.36 × 10^2^–8.24 × 10^4^] pfu/mL, respectively) (Mann-Whitney test, *p* > 0.05). The samples’ epidemiological and laboratory data are summarized in Table 1 (See Table S2 for detailed depiction).

### 3.2. Phylogenetic Analysis

**Figure 1:**
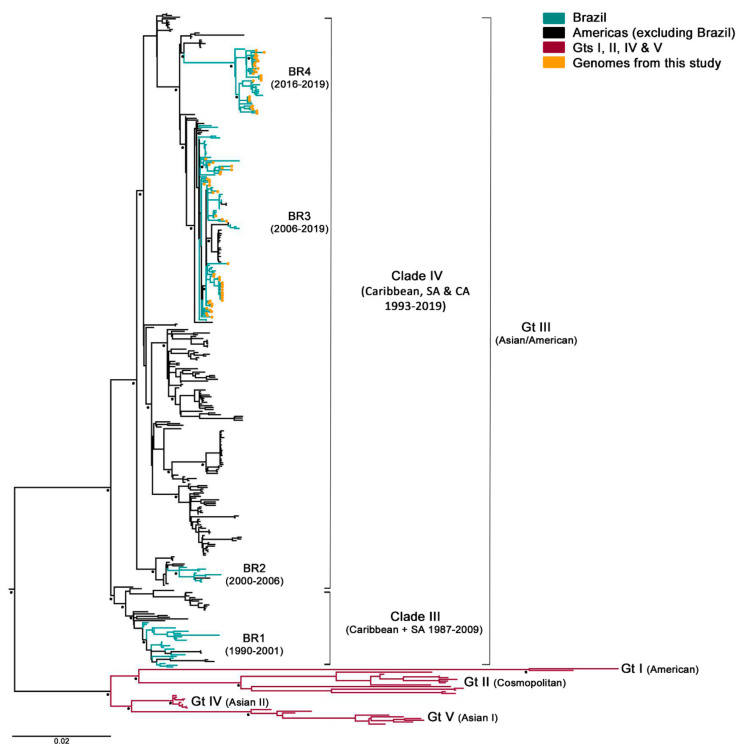
Maximum likelihood phylogenetic tree of DENV-2 polyprotein. It was constructed in RaxML v8.2.8 under GTR + I+G substitution model (General Time Reversible with gamma distribution and invariant sites), and 1000 bootstrap replicates. Main nodes with more than 70% of replicate trees of the bootstrap test for which the taxa clustered together are denoted with a black star. Brazilian sequences obtained in this study are represented in the tree with an orange circle. Gt: genotype; SA: South America; CA: Central America; and BR1-4: Brazilian lineages 1 to 4.

After samples’ deep-sequencing, a full-length viral consensus sequence was obtained for each sample, which were included in a dataset composed of 321 DENV-2 complete genomes retrieved from GenBank. Next, the aligned sequences were phylogenetically analyzed. As expected, all of this cohort’s specimens corresponded to genotype III (formerly known as Asian/American), which is circulating in Brazil since its introduction in 1988–1989. However, samples grouped within two different clusters with 100% bootstrap supported (Figure 1) Brazilian lineage BR3, which corresponded to viral strains circulating after DENV-2 re-introduction in the state of Rio de Janeiro in 2007, and the newly described lineage BR4, which included strains circulating in the country since 2016 [41,42]. Even though BR4 might be a new lineage spreading through Brazil, their representing sequences still grouped within the clade IV of genotype III and was not as genetically diverse from BR3. Additionally, contrary to what was described for BR1 and BR3 [16], no differences were observed in the clinical–epidemiological pattern between BR3 and BR4. Thus, isolates belonging to BR4 lineage were not excluded from this study.

### 3.3. Intrahost Viral Population Structure

DENV-2 genome was next analyzed in-depth, to look for any particular pattern among clinically clustered samples. The median DENV-2 genomic coverage depth was 465.5 (range 8.75–6030) (Table S2). To limit the potential biases introduced by coverage depth variation, ultra-rare variants with frequencies lower than 0.5% were discarded, as well as any presenting considerable strand-bias.

**Figure 2:**
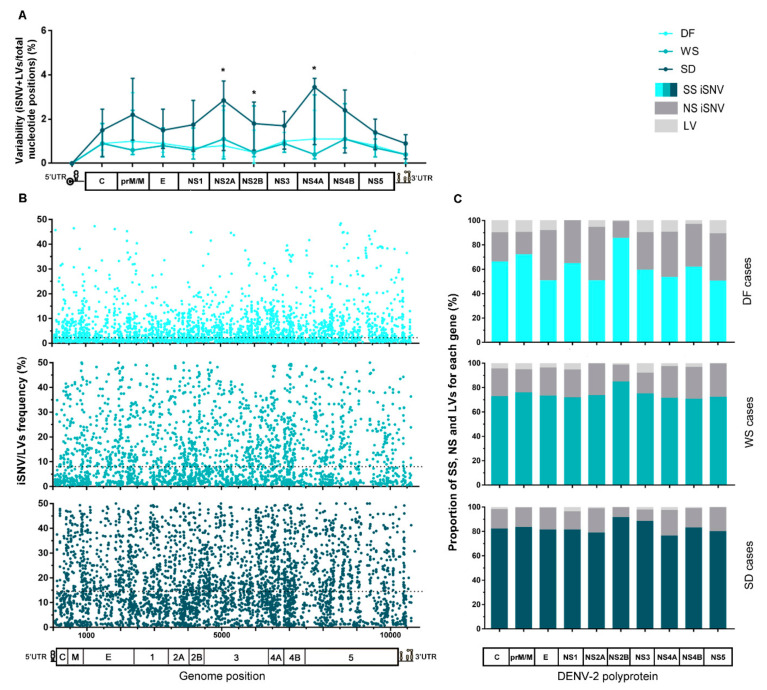

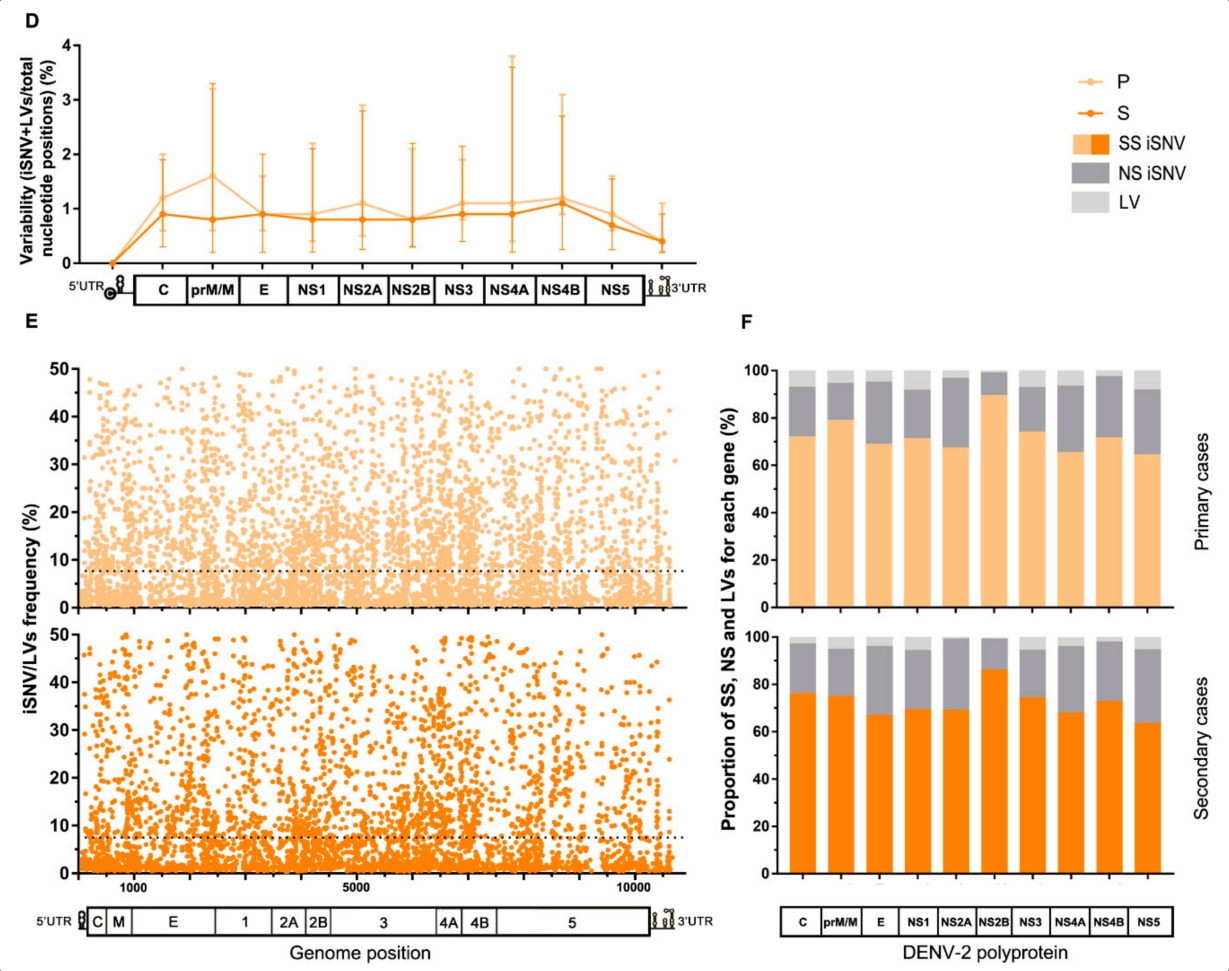
Characteristics of the intrahost viral population of cases grouped by clinical category or immune response. (**A**) Variability along the viral genome according to the patients’ clinical outcome. The total number of iSNVs located in each gene along the viral genome was normalized by each gene’s length (total nucleotide positions) and the median value for each group plotted in the graph, with error bars representing the interquartile ranges. (*) Statistically-supported differences among clinical groups. (**B**) iSNV/LVs frequency distribution along the viral genome according to the clinical category. The dotted line represents the median frequency among all iSNV/LVs found within each group. (**C**) Percentage of synonymous, non-synonymous iSNVs, and LVs for each gene. Clinical categories are represented separately. The total amount of each variant class per gene was summed and then normalized by group size. (**D**–**F**) The same analysis was performed for cases grouped by patients’ immune response. Genome/polyprotein schemes in graphs A, C, D, and F are not scaled to genes real size, as are the schemes in graphs B and E. DF: dengue fever cases, WS: dengue with warning signs, SD: severe dengue cases, P: primary cases, S: secondary cases, SS: synonymous variants, NS: Non-synonymous variants, and LV: insertion/deletion variants.

After mapping sequencing reads of each sample to its corresponding consensus sequence and proceeding with the variant-calling, a total of 9660 intrahost single nucleotide variants (iSNV) and 520 length variants (iLV, representing insertions or deletions) were detected among all samples (see Table S3). To analyze the existence of intrahost diversity patterns, iSNVs and iLVs generated during virus replication were ordered along the viral genome. Then, each gene’s total number was computed for each sample and normalized to the gene’s length to obtain its variability (percent of variations per total nucleotide positions). Finally, median variability for each gene was plotted for the clinically clustered samples (Figure 2A). The same procedure was carried out for the cases that were clustered by immune response (Figure 2D). Even though no correlation seemed to link clinical outcome with immune response in this sampling, it is known that biologically, the immune response is an important factor that might contribute to the severity of the disease [3]. Thus, analysis with samples grouped by this characteristic was performed either way. Overall variability along the complete viral genome was not significantly different between any three clinical groups (Kruskal–Wallis test, *p* > 0.05). However, it did differ notably between the genome regions for SD cases (Kruskal–Wallis test, *p* = 0.0002), with NS2A, NS4A, and NS4B presenting the highest values. Particularly, NS2A and NS2B median variability were higher than that observed for DF (median [IQR]: 2.82% [0.58–3.73] vs. 0.8% [0.3–2.4], *p* = 0.0301 for NS2A, and 1.8% [0.6–2.8] vs. 0.5% [0–1.5], *p* = 0.0259 for NS2B; Mann–Whitney tests). Additionally, NS4A median variability results were also higher than that of WS cases (3.45% [0.85–3.85] vs. 0.4% [0.2–3.1]; Mann–Whitney test, *p* = 0.0471) (Figure 2A).

The intrahost frequency of every SNV/LVs within each host’s viral population was determined during the variant-calling process. The median of the frequency was calculated for all iSNV/LV detected in the total number of samples compounding each category. The lower frequency obtained for the DF cases suggests that viral subpopulations are less represented (median [IQR]: 2.25 [1.16–6.30], while for WS and SD it was 8.00 [2.21–20.00] and 14.49 [7.32–26.50], respectively. Kruskal–Wallis test, *p* < 0.0001). All iSNV/LVs were also combined by genome position and clinical classification (Figure 2B) or immune status (Figure 2E) to assess their frequency distribution along the genome. It was clearly shown that variants within the DF cases were present at minor frequencies indistinctive of genome position (Figure 2B). Furthermore, by observing the effect of each iSNV/LVs over the genome coding region, it was observed that DF presented an overall higher proportion of non-synonymous iSNV (NS-iSNV) and iLVs ($\bar{x}$ ± Sd: 31.75 ± 9.99 and 8.2 ± 3.98) than WS (22.6 ± 4.6 and 4.41 ± 2.76) and SD (15.9 ± 4.3 and 1.63 ± 0.98) (one-way ANOVA test; *p* = 0.0011 and *p* < 0.0001 for NS-iSNV and LV, respectively) (Figure 2C). This observation should be taken together with the shorter infection time of the DF cases and the possibility of many of these variants being deleterious or detrimental to viral fitness. The proportion of synonymous iSNVs (SS-iSNV) for DF cases, which was consequently lower for WS and SD (1-way ANOVA test; *p* = 0.0011) also presented more fluctuation within this group (range: 50.4–85.5), when compared to the other two (70.7–84.7 and 76.4–91.6 for WS and SD, respectively) (Figure 2C). Particularly, E, NS2A, NS4A, and NS5 were the genes that presented the lower proportion of SS-iSNVs (50%); a value that raised to 71–73% and 76–81% within WS and SD, respectively. It is worth noticing that the NS2B gene presented the highest proportion of SS-iSNVs among all coding-genes, irrespective of the patient’s clinical outcome (85% for DF and WS; 92% for SD), which could be likely related to a low tolerance to mutations in the translated protein. 

On the other hand, for samples grouped by patient’s immune response, a similar variability pattern was observed along the entire genome irrespective of the immune response, with no considerable difference for any particular genome region (Mann–Whitney tests, *p* > 0.05) (Figure 2D). As well, no substantial difference was found between the iSNV/LVs’ frequency medians of both groups (median [IQR]: 7.7 [1.94–18.75] for primary, and 7.5 [2.01–17.94] for secondary cases; Mann–Whitney test, *p* > 0.05) (dotted line, Figure 2E), nor in the comparison of the medians for each particular genome region, except for NS2B, where the primary cases presented a discrete higher median frequency (median [IQR]: 10.64 [4.35–19.12] vs. 7.0 [2.83–16.9] for secondary cases; Mann–Whitney test, *p* = 0.0183). Next, when checking the effect of the iSNVs on the coding genes, slightly higher proportions of NS-iSNVs were noticed among the secondary cases for prM/N and NS1 in the first place, followed by NS2B and NS5 (Paired *t*-test; *p* = 0.0132). On the contrary, primary cases showed slightly higher proportions of iLVs in C and NS1 first, followed by NS4A and NS5 (Paired *t*-test; *p* = 0.0022) (Figure 2F). It is interesting to note that C, prM/M, and NS1 are the viral genes on which the immune system’s pressure causes the greatest impact [43,44,45].

**Figure 3:**
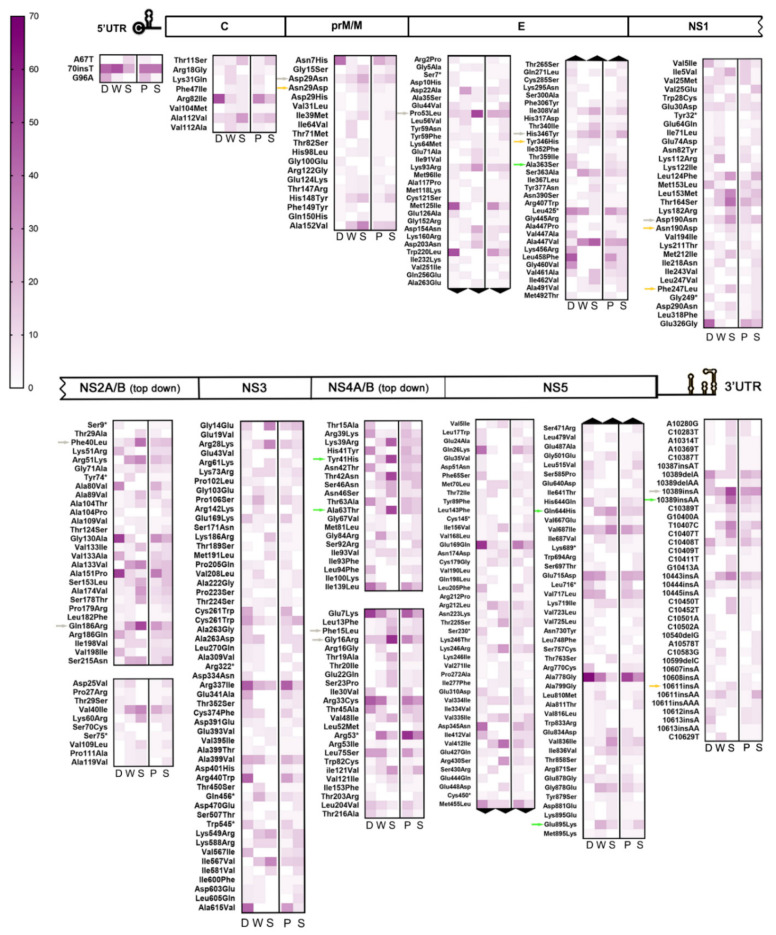
Interhost frequency of iSNV/LVs consistently detected among samples. On this graph, only those repNS-iSNV + UTR-iSNVs found in at least two different samples were considered for analysis. From left to right, F: DF cases, W: WS cases, S: SD cases; *p*: primary infection, and S: secondary infection. Colored-scale indicates the percentage of positive samples, with color intensity and numeric scale increasing with interhost frequency. Colored arrows indicate the most relevant variants within subgroups 2 (green), 3 (orange), and 4 (grey).

Based on the above pieces of evidence, a subsequent detailed analysis of all iSNV/LVs detected in this sampling was carried out. It was observed that from the whole dataset comprising 10,180 intrahost variants, 8948 accounted for 1474 different variants that were consistently repeated among samples, leaving a remaining of 1232 unique variants. After grouping data by clinical outcome, no significant difference was detected for the proportion of unique variants among each group’s samples (one-way ANOVA test, *p* > 0.05; Figure S4). Thus, to look for any mutational pattern that could be correlated with the disease’s clinical outcome, special attention was paid to repetitive NS-iSNVs (repNS-iSNVs), hypothesizing that if minor variants with the potential to alter viral fitness were transmitted, then they could be identified from repetitive patterns among samples. In this regard, and considering that numerous alterations in the UTR regions were indirectly linked to pathogenesis [46], iSNVs located in those regions were added to the repNS-iSNVs repertoire for exhaustive analysis. Variants composing this subset (344 different NS-iSNV + 41 iSNVs located in the UTRs) were grouped according to their genome position and to their clinical group, normalized by group size, and finally plotted on heat maps (Figure 3). A similar analysis was performed on samples classified by the patient’s immune status to try to address whether repNS-iSNVs could arise as a result of immunological pressure (Figure 3). 

Interestingly, 65 repNS-iSNVs + 10 UTR-iSNVs were exclusively found among DF cases (subgroup 1), while 77 repNS-iSNVs + 14 UTR-iSNVs were present only among WS and SD cases (subgroup 2). Of these, 17 repNS-iSNVs + 2 UTR-iSNVs (DF), and 23 repNS-iSNVs + 1 UTR-iSNVs (WS + SD) were highly represented among all samples (*n* ≥ 5). To highlight, 10/17 and 9/23 of the latter represented variants with non-conservative amino acid substitutions (aa), i.e., residues whose physicochemical properties would be altered (Table S5). However, it is also important to mention that intrahost frequency was considerably low for repetitive variants amid DF cases (median: 1%, range: 1–34%), suggesting that they might not be influential for their respective mutant swarms. In the case of subgroup 2, on the contrary, 5 of the repNS-iSNVs with relevant aa substitutions + 1 UTR-iSNV were present amid the WS and SD cases in high intrahost frequencies (median: 21%, range: 1–47%). They are denoted in Figure 3 with green arrows (for further information, see Table S5). In addition, none of these highly represented variants was found exclusively among primary or secondary cases. 

On the other hand, another subgroup of 46 repNS-iSNVs + 10 UTR-iSNVs was also found to be exclusive amid the WS and SD cases (subgroup 3), however, with a lower interhost frequency (*n* = 2–4). This time, the 24/46 variants carried a non-conservative aa substitution (Table S5), and 12 of them were present only amid the primary cases. Additionally, 4 of the repNS-iSNVs with relevant aa substitutions + 1 UTR-iSNV were present in high intrahost frequencies (median: 24%, range: 3–41%). These are highlighted in Figure 3 with orange arrows. 

Finally, a fourth subgroup composed of 58 repNS-iSNVs + 8 UTR-iSNVs stood out from the repetitive variants’ subset because they were found in a high interhost frequency (*n* = 5–19) and in the three clinical categories, but with a marked tendency of increasing/decreasing interhost frequency from the DF to WS + SD cases. Particularly, 22 were highly represented among the DF cases (10–71% vs. 5–37% and 0–17% for WS and SD, respectively), with 5 being located in the UTRs and 11 carrying non-conservative amino acid substitutions. Simultaneously, the remaining 44 were mostly present amid the WS + SD cases (5–32% WS + 11–56% SD vs. 3–19% DF), with 3 located in the UTRs and 15 carrying non-conservative aa substitutions (Table S5). Among the latter, 8 repNS-iSNVs with relevant aa substitutions + 1 UTR-iSNV were found at high intrahost frequencies (median: 16%, range: 1–49%) (Figure 3, grey arrows). This fourth subgroup drew attention because even though these variants did not belong to any particular clinical group, the differences between their interhost frequencies were evident enough. It is also known that depending on the surrounding variants within their respective mutant swarms, they could become relevant [47].

The relevant question here is whether any or all of the above-described variants do actually ascribe the viral particle with more harmful properties, and ultimately whether the improved viral fitness can be correlated to patients’ clinical outcome. However, it was observed that despite the fact they were detected at high inter and intrahost frequency and led to potential relevant residues substitutions in the respective proteins, or changes in the UTRs, for 70% of them (14/20), the inverse iSNV was detected in the other DF cases. For example, while 4 WS + SD cases carried the T3162A-Phe247Leu iSNV in NS1, another DF case carried the A3162T-Leu247Phe iSNV. This means that actually, despite the Leu247 variant being present at a high proportion within the WS + SD viral populations, it was detected at the consensus level (represents the majority of the viral population; allele frequency higher than 50%) in a DF case, which ultimately decreased the chance of that particular variant being associated with a severe clinical outcome. A new approach and further studies would be needed to determine their effect on the viral populations. 

### 3.4. Intrahost Selective Pressure 

**Figure 4:**
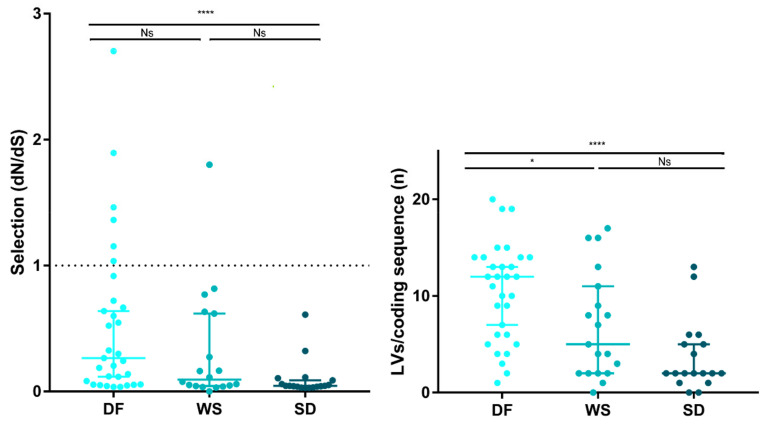
Natural selection strength assessment. The strength of host selection on virus populations was compared between clinical categories. Left: dN/dS ratio over all coding positions. dN/dS ratio of 1, is interpreted as evidence for neutral evolution (dotted line). dN/dS > 1 represents positive selection, while dN/dS < 1 represents a negative (purifying) selection. Right: Number of accumulated iLVs. In all cases, each dot represents a sample and the lines are the median with the IQR. (*) *p* < 0.01; (****) *p* < 0.0001; Ns: not significant.

Natural selection acting on viral populations was assessed by calculating the ratio of non-synonymous (dN) to synonymous (dS) substitutions per coding sequence site (dN/dS), considering both iSNVs and iLVs. Almost all samples (*n* = 61/68; 89.7%) presented dN/dS ratios <1, representing potential evidence that a purifying selection pressure might be taking place to shape the viral populations under study. However, SD cases exhibited dN/dS ratios lower than the DF cases (Mann–Whitney test DF vs. SD; *p* < 0.0001), suggesting that DENV-2 was under a stronger purifying selection in the SD cases (Figure 4), in line with their longer infection times. This is true also for dN and dS when analyzed separately, suggesting that the differences observed between the DF and SD cases were both due to differences in the rates of substitutions at the silent (dS) and non-silent (dN) sites (see Figure S6). Based on the hypothesis that under a prolonged exposure of the virus to the host immune system, only some subpopulations would be positively selected for, we grouped samples by days of symptoms and observed that actually, the median for the dN/dS ratio was the highest for cases with 1 day since the onset of symptoms, with a concomitant decrease up to day 5, correlating with an increased exposure time to the purifying selection (see Figure S6).

Deepening the analysis to check whether the different genes were subjected to a distinct natural selection strength, the dN/dS ratio was also computed for each coding gene within each sample and then analyzed for the samples grouped by clinical classification, immune response, or days of symptoms (see Figure S6). Purifying selection was the strongest in the NS2B and NS3 genes, irrespective of clinical or immune response classification, and days of symptoms. NS2A seemed conspicuously subjected to a stronger negative selection in the WS and SD cases than in the DF cases (Mann–Whitney test, DF vs. WS *p* = 0.0346; DF vs. SD *p* = 0.0134), which was in line with the higher proportion of non-synonymous substitutions found for this latter group. Additionally, a non-statistically supported slight difference was observed in this gene between samples with less than 3 or more than 4 days of symptoms, indicating that selective pressure over this gene might appear later during the infection process. On the other hand, the purifying selection result was weaker for the NS4A gene, which presented the highest dN/dS ratios, independent of the sample classification. Interestingly, and opposite to what was expected, no higher ratios for the highly-immunogenic C, prM/M, E and NS1 coding genes were observed [43,44,45,48]. However, one caveat about these findings, was that the dN/dS variation along the polyprotein could be assessed only for 32/68 samples. For the remaining, the absence of iSNVs in particular genes led to an unviable mathematical calculation of the ratio for that gene, thus, nullifying the analysis for the whole polyprotein. Due to this potential limitation, these last results should be considered cautiously.

Grubaugh et al. proposed that iLVs in the coding sequences are predicted to be deleterious and, consequently, rapidly removed by natural selection [49]. In this context, the strength of the purifying selection was also assessed by analyzing the number of accumulated iLVs per coding sequence (LV/cseq) within the samples grouped by clinical outcome. The SD cases showed the lowest median LV/cseq (median [IQR]: 2 [1.75–5.25]) followed by the WS (5 [2,3,4,5,6,7,8,9,10,11]) and the DF cases (12 [6,7,8,9,10,11,12,13,14]) (Kruskal–Wallis test, *p* = 0.001) (Figure 4). 

## 4. Discussion

Several investigations showed evidence of a strong association between the genetic composition of intrahost viral populations and viral fitness and pathogenicity [9,10,11,12], especially lately that high-coverage and accurate whole-genome sequencing provided proof that fitness and adaptability variations occur without changes in the viral consensus sequence [19,20,50]. However, as recently reviewed by Ko et al., few studies tracked intrahost DENV diversity under epidemiological settings [19,20,50,51]. All of them combined viral genome PCR amplification + deep sequencing experimental strategies. Unlike them, this study employed an amplicon-free in-depth sequencing approach, aiming to reliably reflect the viral genetic diversity at each stage of patient infection, to ultimately assess a correlation with disease severity. To keep that reliability and obtain the patient’s snapshots to analyze DENV-2 intrahost diversity meant resigning to high sequencing coverage depth for samples with low viral load. SD cases, which were shown to present overall lower viral loads, were the critical analytical point of this study. Normalizing the initial RNA load to the limiting sample would lead to an important loss of information in the remaining samples. Thus, with the focus of representing the real DENV-2 intrahost scenario within each patient, and the implementation of several filters after variant-calling to reduce potential error-biasing, we performed the analysis with all samples included in the study (despite the depth variation, due to the initial differences in sample viral load), to look for any particular pattern that could arise from the clinically clustered samples, afterwards. 

In RNA viruses, the generation of variability is determined by the nature of the error-prone replicative RNA polymerase and its replicative rate, while the prevalence of individual mutants depend solely on the fitness of that particular mutant [47]. In our study, we observed that DF cases presented higher proportions of NS-iSNV + iLVs than WS and SD, thus increasing the chance of detrimental non-synonymous mutations, truncated proteins, and defective viral particles (Figure 2C). Two possible explanations are worthy of consideration—(1) in this highly dynamic genetic state of DF cases, the progression of the disease into severity might not be facilitated, or (2) as suggested by Gregory and collaborators the richer viral genetic diversity present in the DF hosts could be beneficial as it would endow the virus population with a greater capacity to adapt to environmental changes as the disease took its course [52]. However, also considering the overall lower intrahost frequencies at which these variants were found (Figure 2B), and the fact these cases represented infection evolutionary times shorter than WS and SD, the most likely explanation would be that differences observed among the clinical groups might be due to distinct periods of intrahost evolution. The process through which intrahost immune pressure shapes DENV-3 diversity during human infections and how this process can be affected by the human host’s immunological background was recently demonstrated [20,50]. Consistently, this would explain why SD cases, submitted to a prolonged and exhaustive purifying selection process (samples taken from day 4 since the onset of symptoms, onwards), accumulated a higher proportion of SS-iSNVs (Figure 2C). 

When analyzing how variability distributed along the viral genome, it stood out that despite the slight differences noticed for three non-structural genes among the clinical categories (NS2A, NS2B, and NS4A), a common trend was stressed for the NS2B gene, regarding its low proportion of NS-iSNVs irrespective of patient’s clinical outcome (Figure 2C). As mentioned before, this can be construed as an activity of the mutated protein poorly supported by the overall viral population, in which case, the NS-iSNVs detected would be expected to be conservative, or variants of recent evolution, which did not yet get the time to be selected for or against. NS2B is a cofactor of the NS3 protease, necessary for the 7-step catalytic processing of the polyprotein. For a full enzymatic activity, NS3 requires the interaction with the NS2B structural loop to comprise residues 49 to 95 [53]. Parameswaran et al. also observed the lowest intrahost diversity along the viral genome to be in this gene [19]. Taken together, this gene could be considered an exciting candidate or target for drugs/vaccine design.

Contrary to the observations from DENV-3 secondary infections [20], we did not find differences in the overall variability between this cohort’s primary and secondary cases. However, secondary infections showed a higher proportion of NS-iSNVs than primary ones, consistent with their shorter infection times (62% presented less than 3 days of symptoms, compared to 38.5% among the primary cases), and likely, with the immune-driven responses. On secondary infections, the heterologous antibodies produced during primary infections could be acting once the virus entered the host, rapidly depurating defective particle product of insertion/deletion variants on the viral genome, which would be consistent with the higher proportion of iLVs found among the primary cases. Additionally, their imposed selective pressure could be the cause of the discrepancies observed for the proportion of NS-iSNV in prM/M and NS1, two highly immunogenic coding genes. To notice, unlike other studies, no distinction could be made between the intrahost diversity of the E gene in the primary and secondary cases [19,20,50]. 

On the other side, if the heterologous antibodies facilitated virus replication, as the ADE theory claimed, and thus, genetic diversity, it would be expected for secondary cases to present a higher viral load compared to primary ones. Actually, no meaningful difference was detected among their medians. Even though it might seem contrary to common findings in which secondary cases tend to develop severe clinical conditions due to ADE, it is important to consider that Katzelnick et al. described a risky window of pre-existing heterologous antibody titer correlated with increased infection severity [3]. It could be likely that some secondary cases of this cohort had pre-existing antibody titers outside the window, and therefore, eluded severe infection. Indeed, only four of the secondary cases developed SD, and in line with this, three of them presented the highest antibody titers obtained by an in-house ELISA assay (Table S7). Either way, conclusions drawn from the analysis of the cases grouped by primary/secondary immune response should be careful, since no statistical correlation was detected in this cohort between patients’ immune response and clinical outcome.

NS-iSNVs + UTR-iSNVs detected amid SD cases could be thought of as the mutants that best adapted to the host environment and the subsequent selective pressure during the 4 or more days of the disease incubation. The UTRs present highly structured domains that mediate viral replication and assist the virus in evading the host innate immune response, hence contributing to determine the infection outcome in the host [reviewed in Reference 46]. Being such constrained areas, it was critical to also assess their presence within this cohort’s cases. Therefore, and to determine whether there was any characteristic mutational pattern emerging after clustering samples by their clinical outcome, NS-iSNVs + UTRs-iSNVs consistently found among the samples were classified according to their presence within each group (Figure 3). The first subset of repetitive variants was found exclusively amid the DF cases and in low intrahost frequencies (subgroup 1, Table S5) suggesting that these variants might not be involved in the severe pathogenesis of DENV-2. It is unclear whether they could represent a disruptive factor for the virus, inducing low fitness and ultimately viral extinction, but this could be a possible explanation for their low frequency (also due to shorter infection times) and prevention of DF case progression into severity. On subgroups 2 and 3, composed of variants found only within the WS + SD cases, 5 and 4 repNS-iSNVs, respectively, were detected in a first instance as relevant variants potentially correlated with SD, due to their inter and intrahost frequencies, and their effect on viral proteins. However, their presence at the consensus level in samples other than these carriers (and mainly DF cases) ruled out this previous hypothesis. On the other hand, this observation could represent a hint that some viral subpopulations could be effectively transmitted after overcoming human–mosquito barriers. Supporting our line of evidence, previous studies with several viral models proved the effective transmission of viral subpopulations, demonstrating that the mutant swarms of viral populations possess minority genomes that reflect those that were dominant at earlier phases of their evolution, serving as a molecular reservoir that is able to swiftly react to selective constraints that were previously experienced by the same population [31,54,55]. From this standpoint, the question arises whether it would be conceivable that these variants were just relevant for transmission but not pathogenesis. Two UTR-iSNVs involving 1–2 nucleotide insertions at positions 10389 and 10611 were also detected in these subgroups (colored arrows on Figure 3; Table S5) but not discarded, as in the previous case. However, 10611insA would not compromise any RNA secondary structure, while 10389insAA is located within stem-loop I, an RNA structure responsible for the generation and accumulation of subgenomic flavivirus RNAs, which play essential roles counteracting antiviral responses in mosquito and human cells [56]. Further studies would be needed to determine the possible effect of this iSNV. Finally, of particular interest were the minor variants detected amongst cases belonging to the three clinical groups (subgroup 4, Table S5). They might be thought of as the variability reservoir capable of conquering human–mosquito transmission bottlenecks, which when exposed to strong purifying selection (as in SD cases), can evolve restoring mutant swarms. It could be likely that repetitive variants of this subgroup might be reflecting this scenario. In turn, 5 of their most relevant repNS-iSNVs were detected at consensus level in up to three WS + SD cases. 

Though the aim was the repetitive variants, unique ones should not be set aside. While it is impossible to trace a mutational pattern from them, their participation in the pathogenesis, either by their potential or by possible epistatic effects cannot be dismissed. Regarding the latter, it is also worthwhile to investigate whether the combined effect of repNS-iSNVs detected here amid the SD cases could elicit severe phenotypes. Epistatic relationships were recently documented for DENV [57,58]. 

Whether there is a potential structural or functional significance for the variants reported here in the DENV-2 genome is an open question. The next step would be to better characterize the effects of these mutations on viral replication and protein interaction, to finally understand the phenotypic traits underlying the viral fitness of these DENV-2 populations and its relation with severe dengue disease. On the other hand, even though this study did not mainly focus on the synonymous SNV repertoire due to its nature, special attention needs to be given in the future to this repertoire, as there is much evidence showing they might not necessarily impair neutral changes [59,60].

The results generated in this study showed that the viral intrahost population structure of these samples varied according to the DENV-2 disease severity, as a straight consequence of the infection time, contributing to understanding the viral dynamics of this virus. 

## Figures and Tables

**Table 1:** Epidemiological information of DENV-2 samples.

Clinical Classification	Number of Cases [*n* (%)]	Gender [M: F (%)]	Age [Mean (Min–Max )]	Location [State (%)]	VL (pfu/mL) [Median (Min–Max )]	Days of Symptoms [Mean (Min–Max)]	Immune Response [*n* (%)]
DF	31 (45.6%)	15:16 (48.4–51.6%)	39.2 (10–65)	RJ (51.6); MG (48.4)	4.31 × 10^4^ (2.47 × 10^2–^8.38 × 10^6^)	2.2 (0–5)	*p* = 17 (54.8%) S = 14 (45.2%)
WS	18 (27.9%)	12:7 (63.2–36.8%)	36.7 (8–85)	RJ (84.2); MG (10.5); SP (5.3)	3.78 × 10^3^ (4.68–1.32 × 10^6^)	2.9 (0–12) *a	*p* = 8 (42.1%) S = 11 (57.9%)
SD	17 (26.5%)	6:12 (33.3–66.7%)	31.7 (7m-88) *a	RJ (77.3); SP (22.2)	1.48 × 10^2^ (2.86–4.94 × 10^5^)	7.1 (4–24) *b	*p* = 14 (77.8%) S = 4 (22.2%)
Total	68	33:35 (48.5–51.5%)	36.6 (7m-88)	RJ (67.6); MG (25.0); SP (7.4)	1.21 × 10^4^ (2.86–8.38 × 10^6^)	3.6 (0–24) *	*p* = 39 (57.4%) S = 29 (42.6%)

DF: dengue fever; WS: dengue with warning signs; SD: severe dengue; M: male; F: female; RJ: state of Rio de Janeiro; SP: state of São Paulo; MG: state of Minas Gerais; VL: viral load; pfu: plaque formation unit; P: primary infection; S: secondary infection; * a: information not determined for one sample; and * b: information not determined for two different samples.

## Data Availability

Publicly available datasets were analyzed in this study. This data can be accessed from the NCBI BioProject: PRJNA541495.

## Supplementary Materials

The following are available online at https://www.mdpi.com/1999-4915/13/2/349/s1, Table S1—Statistical correlations between patient’s clinical outcome and immune response or days of symptoms. Table S2—Clinical, epidemiological, and sequencing sample characteristics. Table S3—iSNV/LVs repertoire of each sample. Figure S4—Percentage of unique variants in mutant swarms of cases grouped by clinical classification. Table S5—Repetitive NS-iSNVs + UTR-iSNVs clustered by subgroups according to their interhost frequency within the clinical categories. Figure S6—Natural selection (dN/dS) assessment for samples grouped by patient’s immune response and days of symptoms. Table S7—Immune response classification. Table S8—GenBank accession numbers of the DENV-2 sequences employed in the phylogenetic analysis.
